# A Short-Term Altrenogest Treatment Post-weaning Followed by Superovulation Reduces Pregnancy Rates and Embryo Production Efficiency in Multiparous Sows

**DOI:** 10.3389/fvets.2021.771573

**Published:** 2021-11-18

**Authors:** Henar Gonzalez-Ramiro, Cristina Cuello, Josep M. Cambra, Alejandro Gonzalez-Plaza, Juan M. Vazquez, Jose L. Vazquez, Heriberto Rodriguez-Martinez, Maria A. Gil, Alejandro Lucas-Sanchez, Inmaculada Parrilla, Emilio A. Martinez

**Affiliations:** ^1^Department of Medicine and Animal Surgery, Faculty of Veterinary Medicine, International Excellence Campus for Higher Education and Research, Institute for Biomedical Research of Murcia, University of Murcia, Murcia, Spain; ^2^Department of Research and Development, Grupo Agropor I+D+I, AIE, Murcia, Spain; ^3^Department of Biomedical & Clinical Sciences (BKV), BKH/Obstetrics & Gynaecology, Faculty of Medicine and Health Sciences, Linköping University, Linköping, Sweden

**Keywords:** Altrenogest, estrus synchronization, superovulation, weaning, embryo transfer, embryo, pig

## Abstract

Although embryo transfer (ET) is a biotechnology ready for the swine industry, there are factors to be solved, the availability of embryo donors as one. Multiparous sows as donors ought to be considered since weaning is a natural and efficient method for estrus synchronization. In addition, superovulation treatments at weaning are effective in increasing the efficiency of donor embryo production. However, ET programs typically require more donors than those available from a single weaning, imposing grouping several weanings to establish a batch for ET. Since short-term administration of Altrenogest is effective in delaying estrus after weaning without effects on ovulation and embryo development, we investigated how Altrenogest combined with superovulation would affect reproductive parameters and embryo quality and quantity of weaned multiparous donor sows. The sows were administered Altrenogest from the day of weaning for 14 (SS-14 group; *N* = 26), 7 (SS-7 group; *N* = 31) and 4 (SS-4 group; *N* = 32) days. The sows were superovulated with eCG 24 h after the last administration of Altrenogest and with hCG at the onset of estrus. Sows not treated with Altrenogest that were superovulated with eCG 24 h post-weaning and hCG at the onset of estrus (SC group; *N* = 37) and sows with natural estrus after weaning (C group; *N* = 34) were used as control groups. The percentage of sows showing estrus within 10 days was not affected by the treatment, but the interval from Altrenogest withdrawal to estrus was longer (*P* < 0.05) in the SS groups than the interval from weaning to estrus in the controls. SS treatments increased (*P* < 0.05) the percentage of sows with ovarian cysts and the development of polycystic ovaries. The pregnancy and the fertilization rates, and the overall embryo production efficiency were also negatively affected by the SS treatments (*P* < 0.05). Interestingly, almost 70% of the structures classified as unfertilized oocytes or degenerated embryos in sows from the SS groups were immature oocytes. In conclusion, although superovulation of weaned sows was highly efficient, short-term administration of Altrenogest in combination with superovulation had negative effects on most of the reproductive parameters assessed, particularly affecting the overall efficiency of pregnancy and embryo production.

## Introduction

Embryo transfer (ET) is atechnology that is in high demand by the swine industry because its commercial use could have unprecedented sanitary, productive, and economic impacts on the pig sector. However, pig ET has been considered impractical for many years, primarily because of the surgical procedures required to obtain and transfer embryos, in addition to the difficulties in preserving embryos from this species. These circumstances have changed considerably in recent years, as recent advances now allow nonsurgical ET with short- (liquid state) and long-term (vitrified) preserved embryos (1–7).

To achieve optimal reproductive outcomes after nonsurgical ET, a high number of embryos (> 25 embryos) must be transferred into each recipient. Although there is a large individual variability in ovulation rates (8, 9), pigs typically ovulate between 15 and 25 oocytes, meaning that the number of donors per recipient would be between 1 and 1.5:1. However, in practice, there are factors that increase this number to 2.5–3.5:1 (10), resulting in an increased cost per ET. One way to reduce this ratio is to superovulate the donor females, which must be synchronized to group the animals efficiently and to facilitate and maximize the effectiveness of embryo collection.

Superovulation with 1,000 IU equine chorionic gonadotropin (eCG) and 750 IU human chorionic gonadotropin (hCG) 24 h post-weaning and at the onset of estrus (72–96 h posteCG), respectively, markedly increase the number of ovulations and the number of viable and transferable embryos, with no effect on fertilization rates or embryo quality (11). Moreover, no differences in fertility or prolificacy were observed after nonsurgical ET of superovulated and control embryos (11, 12).

Primiparous and multiparous sows are an interesting source as embryo donors since most of them undergo a fertile estrus within seven days of weaning. Weaning is therefore an extremely effective natural method of estrus synchronization in sows. However, ET programs often require more donor sows than are available from a single weaning, making it necessary to combine several successive weanings to obtain the number of donor sows sufficient to establish a batch for ET. Several studies have indicated that estrus after weaning can be delayed by short-term administration of the synthetic progestogen Altrenogest (13). This method of synchronization could be very interesting for ET programs. Delaying the onset of estrus by a week or two after weaning would allow the required donor sows to be obtained from several weanings. Since ET programs exclusively use donor sows with a weaning to estrus interval of 3–5 days for logistical reasons (7), Altrenogest treatment for short periods could also be beneficial to reduce the number of unavailable donor sows due to post-weaning estrus outside the appropriate range. This treatment has been shown to be effective in grouping the estrus of weaned sows without adverse effects on ovulation and embryo-fetal development (14, 15). However, the effects of combined short-term synchronization and superovulation treatment on reproductive parameters in weaned sows are still unknown.

The aim of this study was to determine the effects of a short-term protocol for synchronization of estrus combined with conventional superovulation in weaned multiparous sows on estrus and ovulation responses, ovarian characteristics, reproductive parameters, and the quantity and quality of preimplantation embryos produced.

## Materials and Methods

All chemicals used in this experiment were purchased from Sigma-Aldrich Química S.A. (Madrid, Spain) unless otherwise stated.

### Animals

This field study was carried out in a pig production farm (Agropor SL, Murcia, Spain). Multiparous Landrace x Large-White sows with a lactation period of 21–24 days were randomly selected on the day of weaning. Animals were assigned to individual crates under ambient conditions of controlled humidity and temperature facilitated by a forced ventilation system. Duroc boars (2 to 3 years old) housed in a boar station producing seminal doses for artificial insemination (AI) (AIM Iberica, Murcia, Spain) were used as semen donors.

The animals had *ad libitum* access to water and were fed according to their nutritional requirements. The experiments were carried out following the Directive 2010/63/EU on animal experimentation and in accordance with the requests of Spanish legislation in the field of research on the care and use of experimental animals (32/2007, of November 7, and RD 1201/2005, of October 10) for the protection of animals used for experimental and other scientific reasons. The study was reviewed and approved by the Ethics Committee for experiments with animals of the University of Murcia (Code: 486/2018).

### Experimental Design

To group the estrus and superovulate sows weaned on different days, we evaluated the effect of a short-term synchronization treatment combined with superovulation on reproductive parameters and the quality and quantity of the embryos produced. For this, the sows were administered Altrenogest for 14 (SS-14 group; *N* = 26), 7 (SS-7 group; *N* = 31) and 4 (SS-4 group; *N* = 32) days, beginning the treatment on the day of weaning. Sows were superovulated with eCG (Foligon, Intervet, Boxmeer, The Netherlands) 24 h after the last administration of Altrenogest and given hCG (Veterin Corion, Divasa, Farmavic S.A., Barcelona, Spain) at the onset of estrus. Altrenogest nontreated sows superovulated with eCG 24 h after weaning and hCG at the beginning of estrus (SC group; *N* = 37) and sows with post-weaning natural estrus (C group; *N* = 34) were used as control groups. At weaning, healthy sows were selected by body condition (between 2.8 and 3.2 on a five-point scale), parity (between 3 and 7), and reproductive performance (previous farrowing rate and prolificacy > 85% and 10.5 piglets born, respectively), with no differences among the groups.

The interval between the end of synchronization treatment (SS groups) or weaning (SC and C groups) to estrus was assessed. Sows that showed signs of estrus were inseminated and subjected to laparotomy on day 6 (day 0 = onset of estrus) for examination of their ovaries and the collection of embryos. During laparotomy, the number of corpora lutea and the number and size of ovarian cysts in each ovary were assessed. After collecting the uterine washings, the number of viable embryos, unfertilized oocytes, degenerated embryos, and the stage of embryonic development were also recorded. Finally, the recovery rates (total number of structures collected divided by the total number of corpora lutea present) and fertilization rates (total number of viable embryos divided by the total number of structures collected) were determined. To determine the quality of the embryos, the inner cell mass (ICM) and trophectoderm (TE) cell ratio, apoptosis index and cryotolerance were assessed. Sows that showed no signs of estrus 10 days after the end of Altrenogest treatment or weaning also underwent laparotomy to evaluate the status of their ovaries.

In each replicate, 25 sows (*N* = 5 sows per group) were used, and these sows were all inseminated with seminal doses from the same boar. This experiment was repeated six times to evaluate a total of 160 sows (26 to 37 sows per group).

### Hormonal Treatments, Estrus Detection and Artificial Insemination

Sows were synchronized by oral administration of Altrenogest (Regumate®, Merck Sharp & Dohme Animal Health, S.L., Salamanca, Spain) at a dose of 20 mg per sow per day. Sows were superovulated with 1,000 IU eCG (i.m.). Detection of estrus was performed by trained personnel in the presence of vasectomized boars once daily, starting on the day of the end of synchronization treatment or the day of weaning. At the onset of estrus, the sows were treated with 750 IU hCG (i.m.) and inseminated. Standard AIs were carried out 6 and 24 h after the onset of estrus with fresh or stored for 24 h at 18°C AI doses containing 3 × 10^9^ spermatozoa extended in 90 mL of BTS extender (Beltsville thawing solution) (16). Motility and sperm morpho-anomalies of the AI doses were >70 and <15%, respectively, at the time of insemination.

### Surgical Procedure and Embryo Recovery

Surgical and embryo recovery procedures were performed as previously reported (17). Briefly, sows were sedated and then anesthetized with azaperone (i.m.; 2 mg/kg body weight; Stresnil^®^, Landegger Strasse, Austria) and sodium thiopental (i.v.; 7 mg/kg body weight, intravenous; B. Braun VetCare SA, Barcelona, Spain), respectively. Anesthesia was maintained with isoflurane (3–5%; IsoFlo^®^, Madrid, Spain). After exposure of the reproductive tract, the ovaries were examined to determine their response to the hormone treatments. The ovulatory response of the sows was assessed by counting the corpora lutea in both ovaries. The presence of follicular cysts (ovarian structures filled with a transparent fluid without ovulatory signs and with a diameter of > 1 cm at the time of laparotomy) and polycystic ovaries (ovaries with more than four large follicular cysts, without visible corpora lutea formations) was recorded in each sow. Embryos were recovered by washing the tip of each uterine horn with 30 mL of modified Tyrode's lactate-HEPES-polyvinyl alcohol (THP) medium (7, 18) at 37°C, and the number of unfertilized oocytes and viable and degenerated embryos was recorded for each sow.

### Evaluation of Oocytes and Degenerated Embryos

For nuclear assessment, unfertilized oocytes and degenerated embryos were fixed, stained with lacmoid, and assessed microscopically, as previously reported (19). Oocytes with chromatin enclosed by a nuclear membrane were considered immature oocytes at the germinal vesicle (GV) stage, and oocytes with chromosomes organized in metaphase and with the presence of an extruded first polar body were considered mature oocytes at the metaphase II stage. Degenerated structures with multiple stained nuclei were considered degenerate embryos.

### Embryo Quality Assessment

Embryo quality evaluation was conducted by studying the morphology, differential staining (number of ICM and TE cells), apoptosis and cryotolerance.

#### Morphology

The embryos were assessed for their stage of development and quality under a stereomicroscope. Unicellular eggs were considered oocytes. Insufficiently or poorly developed embryos were considered degenerate embryos. Embryos that exhibited an appropriate developmental stage for age (day 6: morulae and blastocysts) and good or excellent morphology following the International Embryo Transfer Society criteria (20) were considered viable. These criteria included embryos with a spherical shape, an intact zona pellucida, no or few extruded blastomeres, compact blastomeres (for morulae), or a discernible blastocoele and ICM and TE cells (for blastocysts).

#### Cell Number in the Blastocysts: Differential Staining

Total cell number (TCN) and the numbers of ICM and TE cells were counted in the blastocysts following an immunofluorescence procedure (21). Briefly, viable embryos were fixed in paraformaldehyde, permeabilized with Triton X-100 and Tween 20 and incubated sequentially in HCl and Tris-HCl. Embryos were then washed, placed in blocking solution, and washed again before incubation with a primary antibody (CDX-2) that specifically binds to TE cells. Next, the embryos were washed, incubated with Alexa Fluor^®^ 568-donkey anti-mouse IgG secondary antibody, transferred to microdroplets containing Vectashield-Hoechst 33342 solution, and examined under a fluorescence microscope to count the Hoechst-stained nuclei (TCN; blue fluorescence) and the TE cells (red fluorescence) at excitation wavelengths of 330–380 and 536 nm, respectively. The ICM was calculated by subtracting the number of TE cells from the TCN.

#### Cellular Apoptosis

Apoptosis was determined in morulae using the APO-BrdUTM TUNEL Assay Kit (A23210; Invitrogen, Oregon, USA) as previously reported (22). Briefly, viable embryos were fixed in paraformaldehyde and permeabilized with Triton X-100 and Tween 20. Positive control embryos were incubated in Dnase I. Then, control and experimental morulae were transferred to PBS-BSA medium containing Tween 20 and incubated in TUNEL reaction medium. Finally, the embryos were washed, placed in microdroplets of Vectashield-Hoechst 33342 solution, and examined by fluorescence microscopy. Cells with green fluorescent nuclei (465 to 495 nm excitation wavelength) were classified as TUNEL+. The proportion of TUNEL+ cells to total Hoechst-stained nuclei (blue fluorescence; 330 to 380 nm excitation wavelength) was the apoptotic index.

#### Cryotolerance

Cryotolerance was used as a marker of embryo quality and as a potential predictor of embryo developmental ability. Morula stage embryos from each treatment group were vitrified within 3 h of embryo recovery using the procedure described by Cuello et al., 2016 (5). Briefly, embryos were washed in THP medium and incubated in THP containing 7.5% dimethyl sulfoxide (DMSO) and 7.5% ethylene glycol (EG) and then in THP containing 16% DMSO, 16% EG and 0.4 M sucrose. Groups of 5–6 embryos were loaded into super open pulled straws and placed in liquid nitrogen. After storage (1 month), the embryos were warmed in THP medium supplemented with 0.13 M sucrose and cultured for 24 h in NCSU-23 medium (23) containing bovine serum albumin (0.4%) and fetal calf serum (10%) at 38.5°C in 5% CO_2_ in air. The embryos were examined morphologically to determine their viability and developmental stage. Morulae that reached the blastocyst stage with excellent or good morphology at the end of culture were considered viable. The survival rate was defined as the proportion of postwarming viable embryos out of the total number of embryos cultured.

### Statistics

Statistical analysis was performed using the statistical package IBM SPSS 24.0 (IBM, Chicago, IL, USA). The chi-square test with Yates correction when necessary was used for comparisons of the percentage data. Continuous variables were analyzed using the Shapiro-Wilk test to check the normality assumption and compared with ANOVA. The Bonferroni test was performed when necessary for *post hoc* analysis. For statistical analysis, the embryo development stage was scored subjectively on a scale of 1–4 as follows: 1, morula; 2, early blastocyst; 3, blastocyst; and 4, hatching or hatched blastocyst. The end of Altrenogest treatment or weaning to estrus intervals and the embryo development stage were compared with the nonparametric Kruskal-Wallis test, and when necessary, two-by-two comparisons were performed with the Mann–Whitney *U* test. A *P* < 0.05 was considered significantly different. All data are expressed as the mean ± standard deviation (SD) and percentages.

## Results

**Figure 1:**
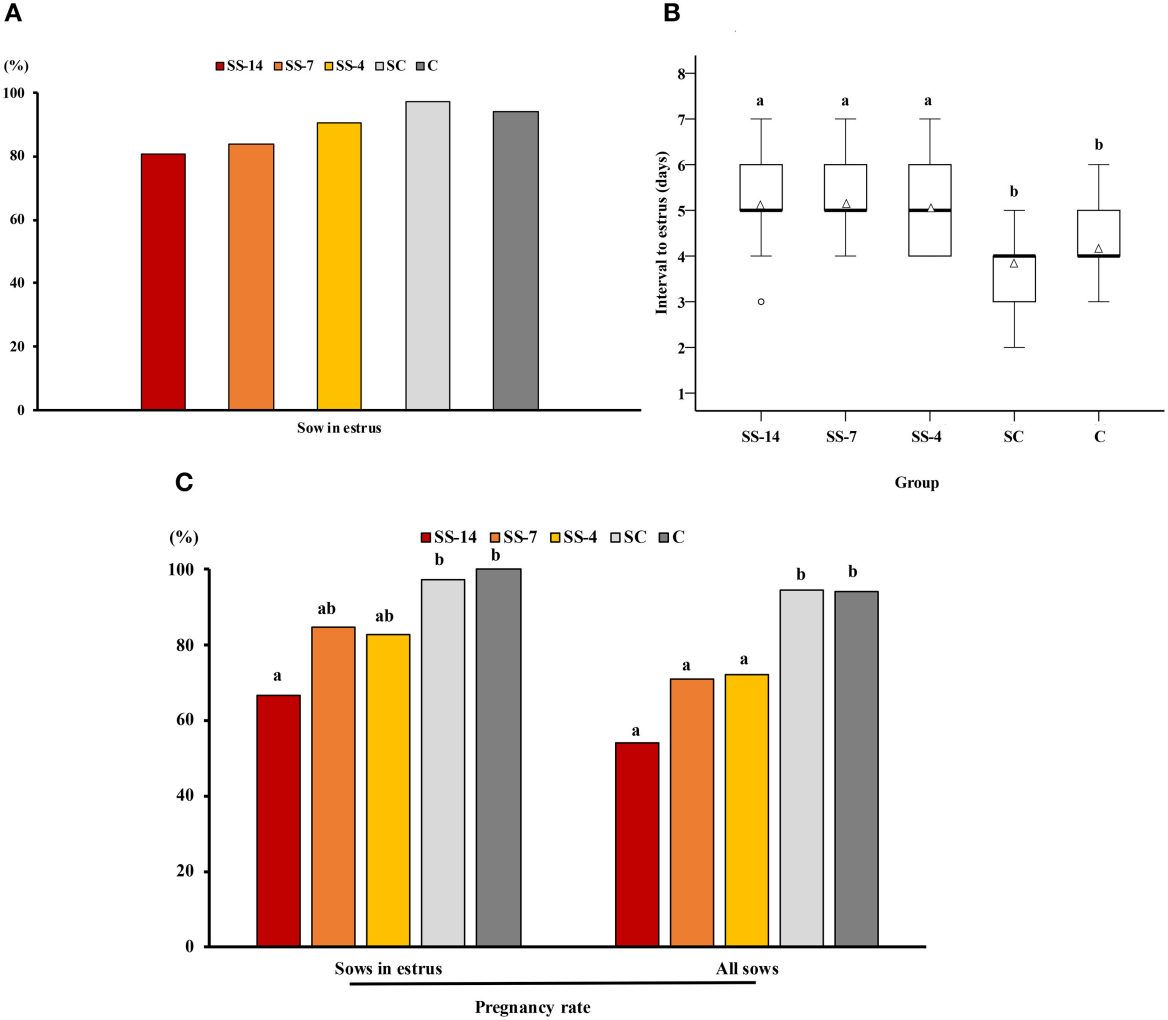
Occurrence of estrus and pregnancy rates in weaned sows superovulated after Altrenogest treatment for 14 (SS-14 group; *N* = 26), 7 (SS-7 group; *N* = 31) and 4 (SS-4 group; *N* = 32) days. Superovulated weaned sows without prior Altrenogest treatment and weaned sows with natural estrus were used as controls (SC group, *N* = 37 and C group, *N* = 34, respectively). **(A)** Percentage of sows in estrus within 10 days of the end of treatment or weaning. **(B)** Box plots showing the interval from the last Altrenogest feeding (SS-14, SS-7, and SS-4 groups) or weaning (SC and C groups) to the onset of estrus. Values are given as medians (thick lines) and interquartile ranges (boxes, Q1–Q3); triangles and circles represent the mean and outliers, respectively. ^a,b^ Different letters indicate significant differences (*p* < 0.01). **(C)** Pregnancy rates 6 days after the onset of estrus in sows from the different groups. Sows with at least four viable embryos were considered potentially pregnant. The pregnancy rate was calculated as the ratio of the number of pregnant sows to the number of sows in estrus or to the total number of sows used. ^a,b^ Different letters within each variable indicate differences (*p* < 0.05).

There was a significant effect of treatment on the interval from Altrenogest withdrawal (SS groups) or from weaning (SC and C groups) to estrus and on the potential pregnancy rate relative to sows that showed estrus or relative to all sows in each group. The percentage of sows that showed estrus within 10 days was not statistically affected by the treatment but ranged from 80.8 to 87.5% in the SS groups and 93.7–94.1% in the control groups (Figure 1A). The interval from weaning to estrus was shorter (*P* < 0.05) for the control sows (SC and C groups) (3.8 ± 0.7 and 4.2 ± 0.7 days, respectively) than the interval from Altrenogest withdrawal to estrus (5.2 ± 1.1, 5.3 ± 0.6, and 5.1 ± 0.8 days, for SS-14, SS-7, and SS-4 groups, respectively) (Figure 1B). The estimated pregnancy rate in sows that showed estrus was affected by the duration of Altrenogest treatment. Fewer (*P* < 0.01) sows from the SS-14 group were pregnant on day 6 of the cycle (66.7%) compared with the control groups (97.2 and 96.9% for SC and C groups, respectively) (Figure 1C). Overall, the estimated pregnancy efficiency, i.e., the percentage of pregnant sows relative to the total number of weaned sows in each group, was negatively affected by the Altrenogest treatment. The efficiency was <75% for SS sows (range 53.8–73.3%), a percentage that was significantly lower (*P* < 0.05) than for control sows (94.6 and 91.2% for the SC and C groups, respectively) (Figure 1C).

**Figure 2:**
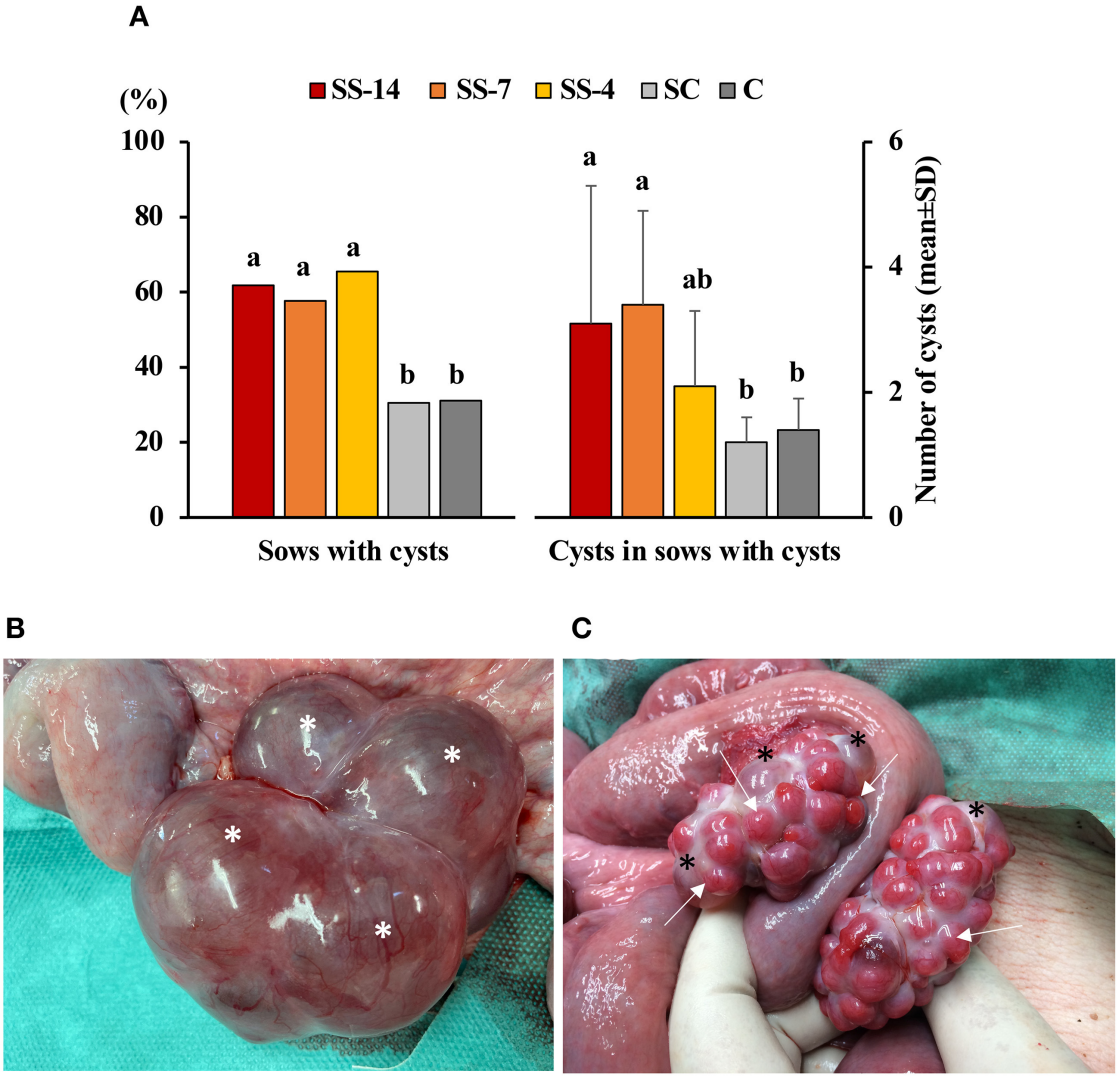
Reproductive disorders in weaned sows superovulated after Altrenogest treatment for 14 (SS-14 group; *N* = 26), 7 (SS-7 group; *N* = 31) and 4 (SS-4 group; *N* = 32) days. Superovulated weaned sows without prior Altrenogest treatment and weaned sows with natural estrus were used as controls (SC; *N* = 37 group and C; *N* = 34, respectively). **(A)** Percentage of sows with cysts and the number of cysts per sow in sows that showed signs of estrus after SS treatments or weaning. ^a,b^ Different letters within each variable indicate differences (*p* < 0.05). **(B)** Representative images of a polycystic ovary at day 6 after the onset of estrus with at least four large follicular cysts (asterisks) and the absence of corpora lutea. **(C)** Representative images of ovaries with numerous corpora lutea (arrows) and several small follicular cysts (asterisks).

The frequency of vaginal discharge and polycystic ovaries in sows with or without estrus after the end of treatments or weaning is shown in Table 1. Vaginal discharge was observed in some sows that showed estrus regardless of treatment. In contrast, synchronization-superovulation treatment affected the development of polycystic ovaries, which were observed only in sows from the three SS groups. None of these sows had corpora lutea on their ovaries, and most cysts were larger than 2 cm. While all control sows and sows from the SS-4 group that showed no signs of estrus had functional ovaries, with the presence of multiple corpora lutea and no alterations of the oviducts or uterine horns, 60% of the SS-14 and SS-7 sows had reproductive abnormalities in the form of vaginal discharge and/or polycystic ovaries. The percentage of sows with ovarian cysts and the number of ovarian cysts per sow in sows that showed estrus after the treatments are shown in Figure 2A. Synchronization-superovulation treatment not only increased (*P* < 0.05) the percentage of sows with ovarian cysts (range of 57.7–65.5%) compared with control sows (~30%) but also increased (*P* < 0.05) the number of ovarian cysts per sow (3.1 ± 2.2 and 3.4 ± 1.5 in SS-14 and SS-7 sows, respectively, and 1.2 ± 0.4 and 1.4 ± 0.5 in SC and C control sows, respectively). Representative images of polycystic ovaries and ovaries with numerous corpora lutea are shown in Figures 2B,C, respectively.

**Table 1:** Incidence of vaginal discharge and polycystic ovaries in sows that did or did not show signs of estrus within 10 days of the end of treatment or weaning.

**Treatment**	**Sows exhibiting estrus**			**Sows not exhibiting estrus**			
	**N**	**Vaginal discharge (N; %)**	**Polycystic ovaries (N; %)**	**N**	**Vaginal discharge (N; %)**	**Polycystic ovaries (N; %)**	**Ovaries with corpora lutea (N; %)**
SS-14	21	0 (0.0)	4 (19.0)^a^	5	1 (20.0)	2 (40.0)	2 (40.0)
SS-7	26	1 (3.8)	1 (3.8)^ab^	5	0 (0.0)	3 (60.0)	2 (40.0)
SS-4	29	2 (6.9)	2 (6.9)^ab^	3	0 (0.0)	0 (0.0)	3 (100.0)
SC	36	0 (0.0)	0 (0.0)^b^	1	0 (0.0)	0 (0.0)	3 (100.0)
C	32	1 (3.1)	0 (0.0)^b^	2	0 (0.0)	0 (0.0)	3 (100.0)

a,b *Different superscripts in the same column indicate differences (p < 0.05)*.

**Figure 3:**
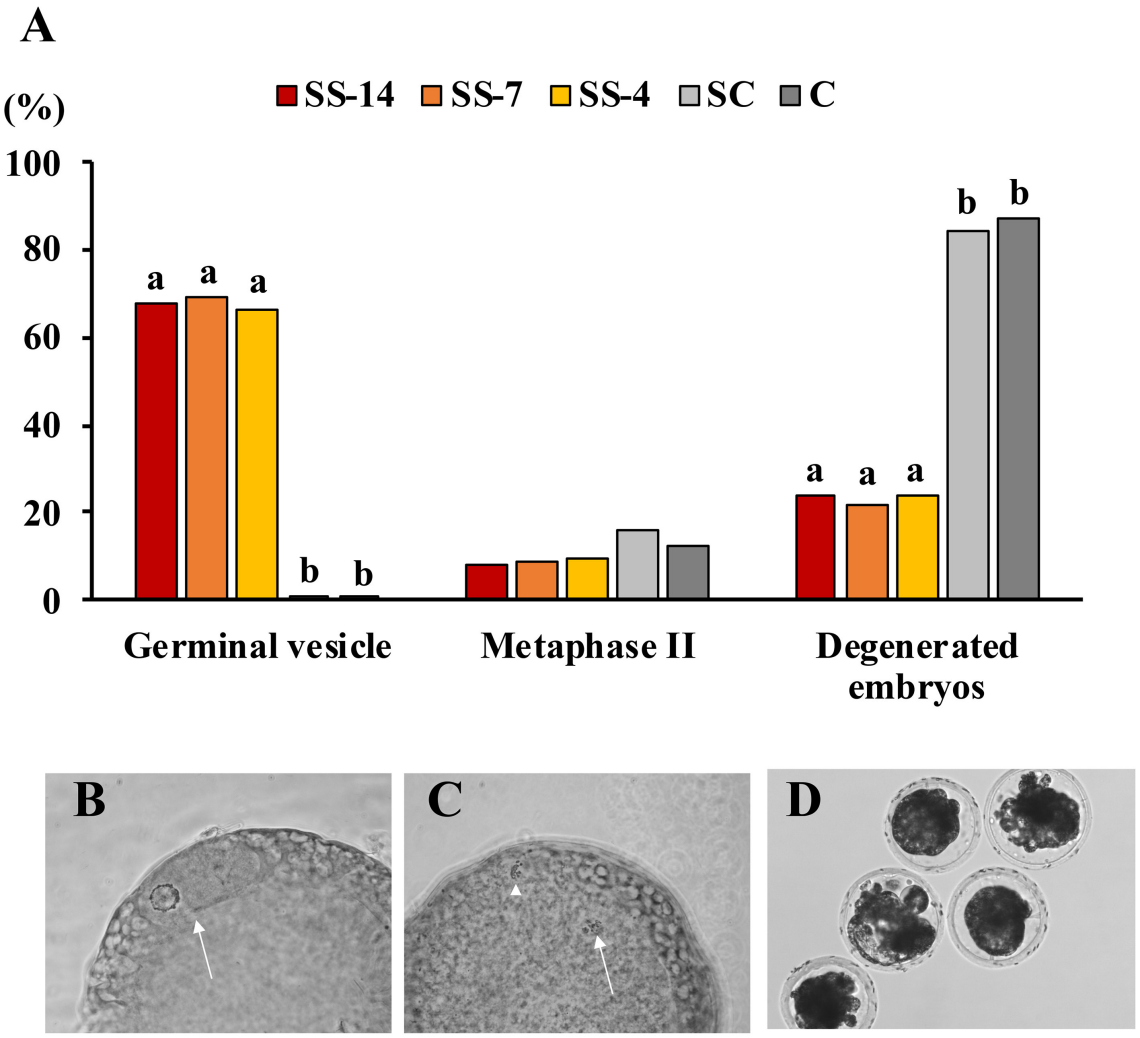
**(A)** Findings of oocyte immaturity in structures classified as unfertilized oocytes and degenerated embryos recovered from weaned sows superovulated after treatment with Altrenogest. The number of structures evaluated was 25, 23, 21, 19, and 16 for groups SS-14, SS-7, SS-4, SC, and C, respectively. ^a,b^ Different letters within each variable indicate significant differences (*p* < 0.01). **(B)** Unfertilized immature oocytes at the germinal vesicle stage (arrow). **(C)** Unfertilized mature oocyte with chromosomes at metaphase stage II (arrow) and the first polar body (arrowhead). **(D)** Embryos classified as degenerate showed irregular morphology and a low cell number after staining with Hoechst 33342.

Fertilization parameters assessed in sows from the five groups are shown in Table 2. The ovulatory response was similar in the hormonally treated groups (range: 28.5 ± 6.5 to 34.1 ± 10.7 corpora lutea) and higher (*P* < 0.05) than in the nontreated C sows (22.2 ± 4.3 corpora lutea). There were no differences in embryo recovery rates (range: 84.0 ± 16.4 to 87.1 ± 14.5%) between groups. The mean number of recovered viable embryos (compacted morulae and unhatched blastocysts) was higher (*P* < 0.05) in the SC group (26.4 ± 6.1) than in the SS and C groups (~19.0). However, the developmental stage of these embryos was similar among the groups. There were differences between groups in the mean number of oocytes and degenerated embryos, which was almost 5-fold higher (*P* < 0.05) in the SS groups (range: 6.3 ± 5.6–7.4 ± 6.4) than in the control groups (1.9 ± 2.3 and 1.4 ± 1.4 for SC and C groups, respectively). Fertilization rates in pregnant sows were almost 20 percentage points lower (*P* < 0.05) (range: 72.5 ± 22.7–74.4 ± 18.3%) in sows of the SS groups than in control sows (93.5 ± 7.8 and 92.4 ± 7.1 for SC and C groups, respectively). The overall efficiency of embryo production, i.e., the mean number of viable embryos obtained from the total number of sows in each treatment, was similar in the SS groups (range: 10.0 ± 11.3–14.7 ± 11.0) and the untreated C group (16.0 ± 7.6) but much lower than in the SC sows (25.0 ± 8.4). The distribution of collected structures classified as oocytes or degenerated embryos after staining and evaluation is shown in Figures 3A–D. In sows from the SS groups, 68.1% (range: 66.7–69.6%), 8.7% (range: 8.0–9.5%), and 23.2% (range: 21.7–24.0%) of these structures were immature oocytes at the GV stage, mature oocytes at the metaphase II stage and degenerated embryos, respectively. These data were very different (*P* < 0.05) in sows from the control groups, where the percentage of immature oocytes was 0.0% and the percentage of degenerated embryos was 85.8%.

**Table 2:** Effects of combined treatments for estrus synchronization and superovulation on reproductive parameters in weaned sows.

	**Treatment**				
	**SS-14**	**SS-7**	**SS-4**	**SC**	**C**
Sows (n)	26	31	32	37	34
Corpora lutea^*^	29.9 ± 5.4^a^	34.1 ± 10.7^a^	28.5 ± 6.5^a^	32.9 ± 7.3^a^	22.2 ± 4.3^b^
Recovery rate (%)^*^	84.9 ± 13.4	84.0 ± 16.4	85.5 ± 22.3	86.9 ± 14.7	87.1 ± 14.5
Viable embryos collected^**^	18.6 ± 8.7^a^	19.7 ± 7.9^a^	18.5 ± 6.2^a^	26.4 ± 6.1^b^	18.1 ± 5.0^a^
Oocytes/degenerated embryos^**^	6.4 ± 8.1^a^	7.4 ± 6.4^a^	6.3 ± 5.6^a^	1.9 ± 2.3^b^	1.4 ± 1.4^b^
Fertilization rate (%)^**^	74.4 ± 27.8^a^	72.5 ± 22.7^a^	74.4 ± 18.3^a^	93.5 ± 7.8^b^	92.4 ± 7.1^b^
Developmental stage (1–4)^&^	1.8 ± 0.9	1.8 ± 1.0	2.1 ± 1.1	1.7 ± 0.9	2.0 ± 1.0
Efficiency of embryo production^#^	10.0 ± 11.3^a^	14.7 ± 11.0^a^	13.5 ± 9.3^a^	25.0 ± 8.4^b^	16.0 ± 7.6^a^

* *In relation to the total sows in estrus*.

** *In relation to the total pregnant sows*.

& *The developmental stage was scored according to the following classes: 1: morulae; 2: early blastocysts; 3: blastocysts; 4: hatching or hatched blastocysts*.

# *Mean of viable embryos collected from the total sows used*.

a,b *Different superscripts in the same row indicate differences (p < 0.05). Values are given as the mean and SD*.

**Figure 4:**
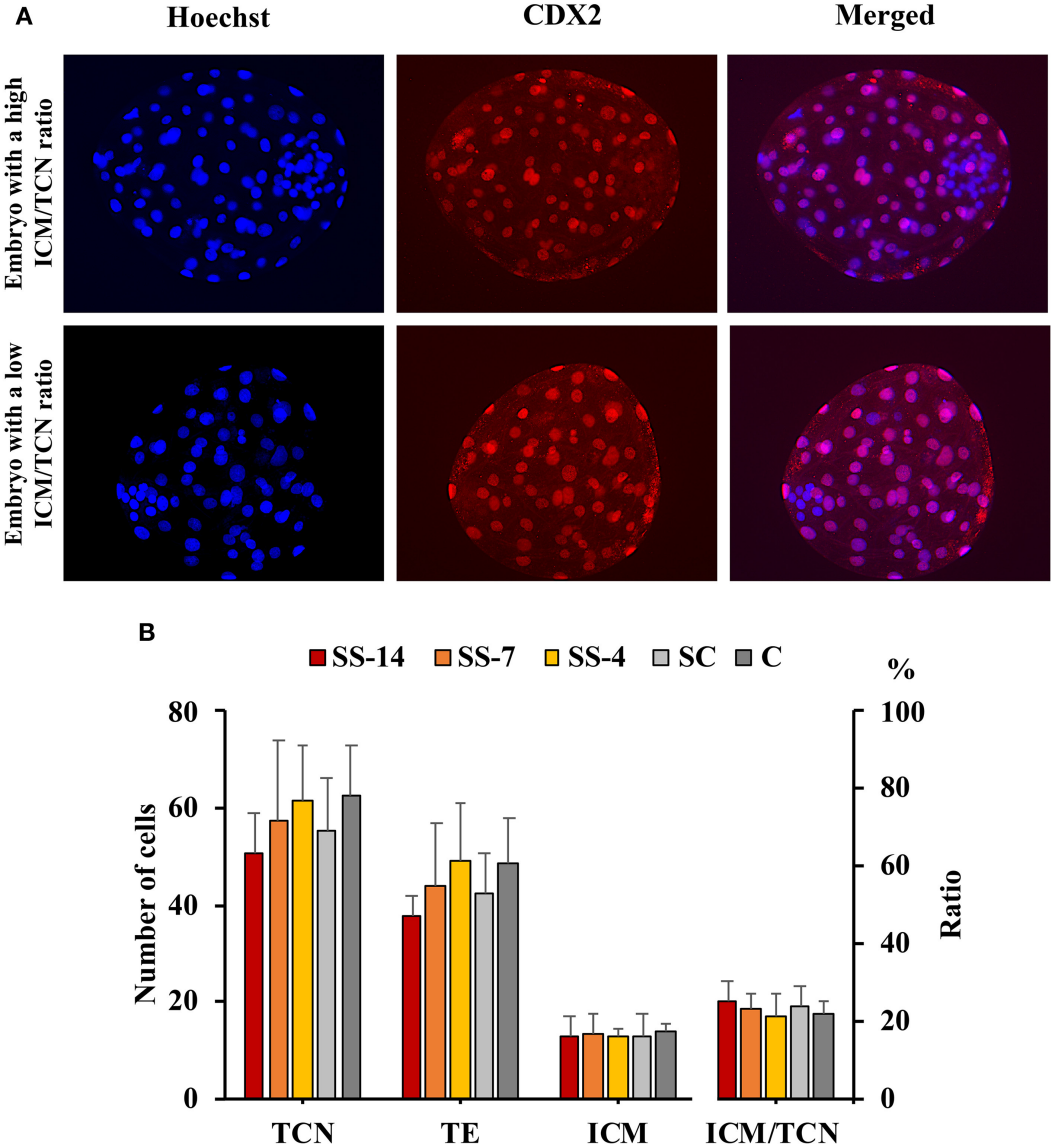
Differential staining of embryos at the blastocyst stage. **(A)** Hoechst-stained nuclei of all blastomeres (blue) and anti-CDX2-stained trophectoderm (TE) cells (red). Merged images show inner cell mass (ICM) and TE cells with blue and pink-red fluorescence, respectively. **(B)** Total cell number (TCN) and ICM/TCN ratio in blastocysts collected at day 6 after the onset of estrus from weaned sows superovulated after Altrenogest treatment for 14 (SS-14 group; *N* = 10), 7 (SS-7 group; *N* = 12) and 4 (SS-4 group; *N* = 12) days. Superovulated weaned sows without previous Altrenogest treatment and weaned sows with natural estrus were used as controls (SC, *N* = 14; and C, *N* = 12).

**Figure 5:**
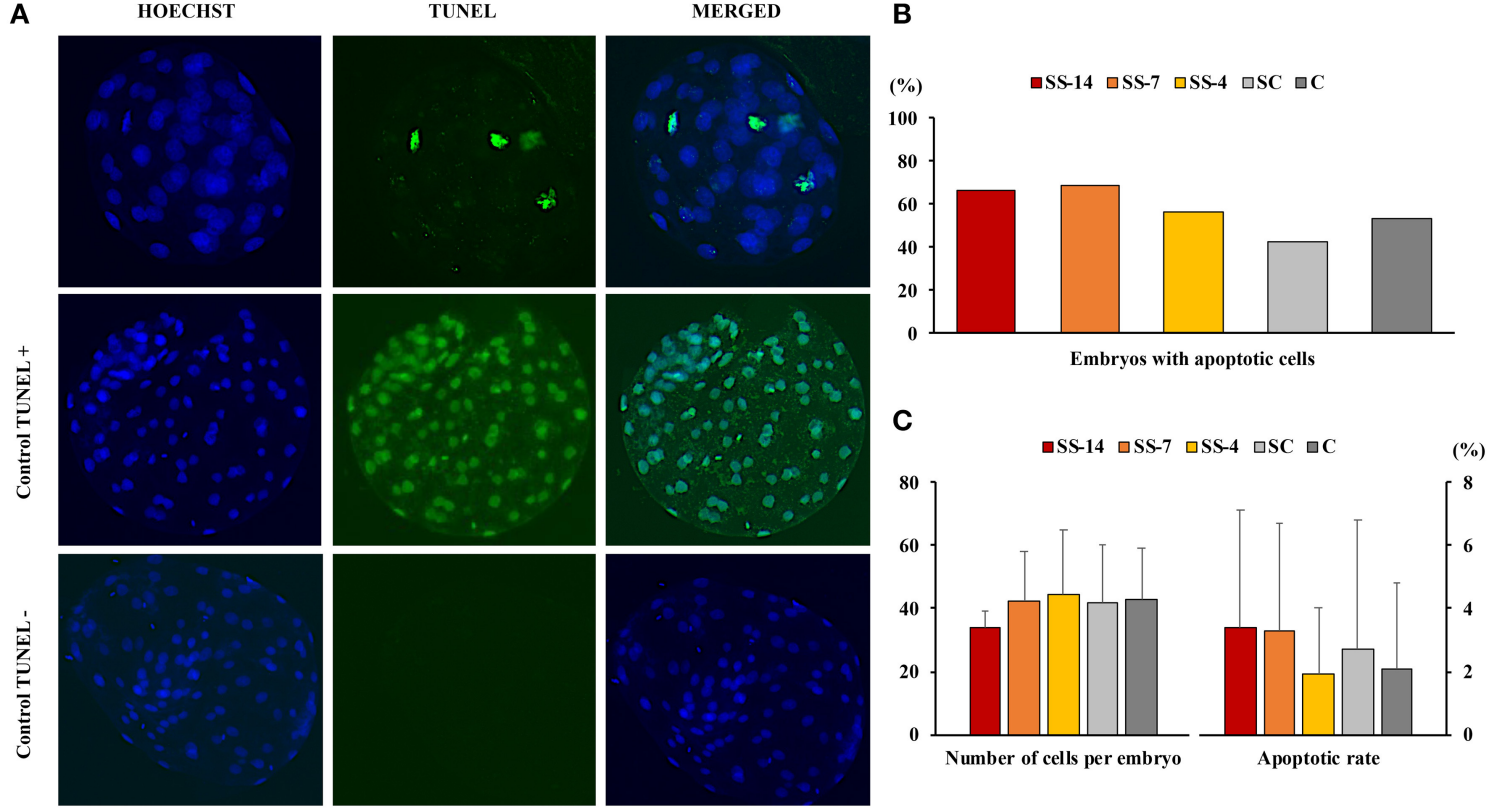
Detection of apoptosis in embryos at the morula stage collected on day 6 after the onset of estrus in weaned sows superovulated after Altrenogest treatment for 14 (SS-14 group; *N* = 15), 7 (SS-7 group; *N* = 16) and 4 (SS-4 group; *N* = 16) days. Superovulated weaned sows without prior Altrenogest treatment and weaned sows with natural estrus were used as controls (SC, *N* = 17; and C, *N* = 15 groups, respectively). **(A)** Representative fluorescence images of the TUNEL assay (including TUNEL-positive and TUNEL-negative controls). The blue Hoechst signal (left), green TUNEL staining (middle), and a merged image (right) are shown. **(B)** Percentage of morulae with apoptotic cells. **(C)** Number of blastomeres and apoptosis rates in embryos from the different groups.

**Figure 6:**
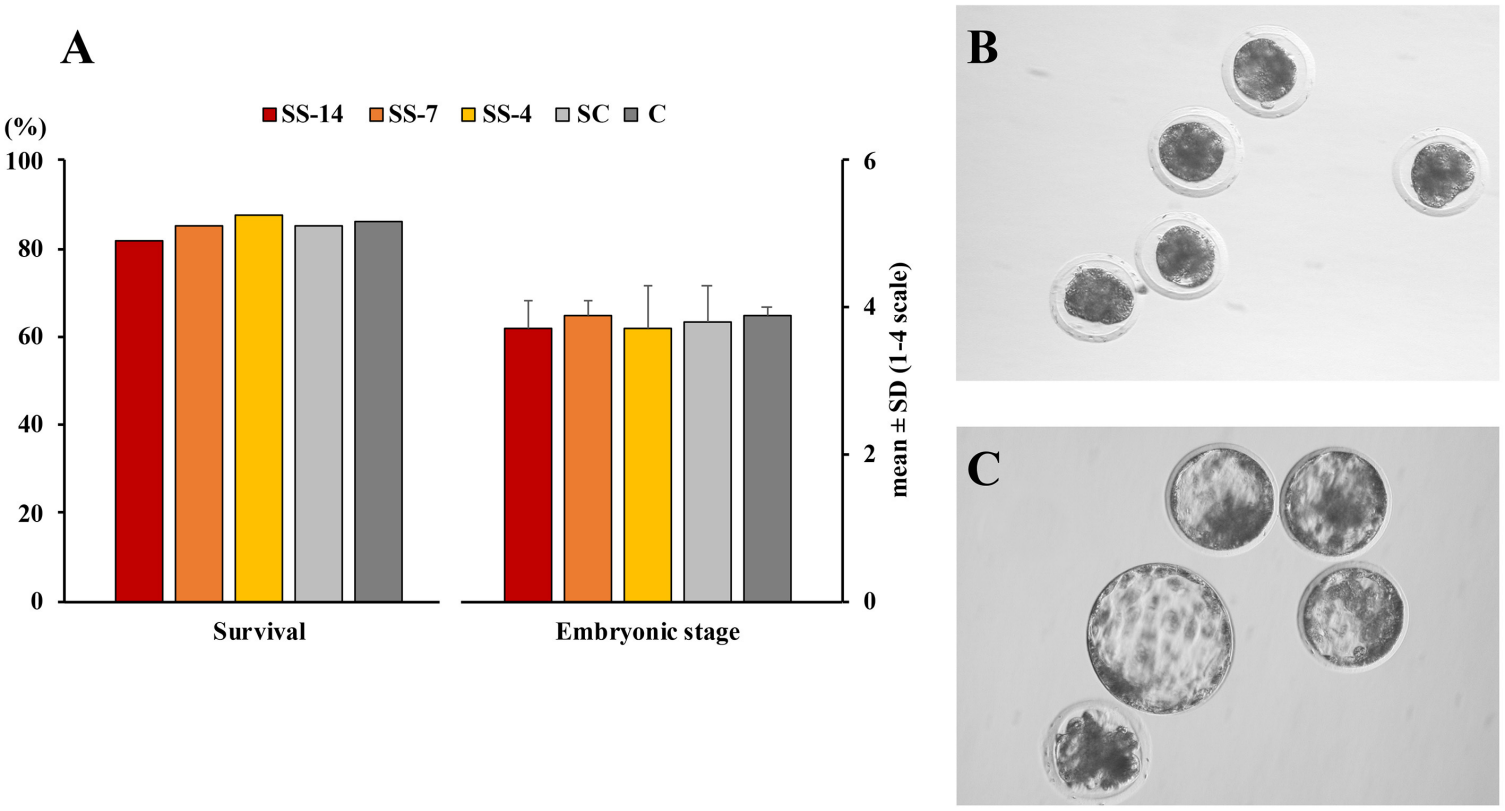
Cryosurvival and embryo development after vitrification and warming. **(A)** *In vitro* survival and embryonic developmental stage after 24 h culture of vitrified and warmed morulae. Fresh morulae were collected from weaned sows superovulated after Altrenogest treatment for 14 (SS-14 group; *N* = 27), 7 (SS-7 group; *N* = 60) and 4 (SS-4 group; *N* = 65) days. Superovulated weaned sows without previous Altrenogest treatment and weaned sows with natural estrus were used as controls (SC, *N* = 68; and C; *N* = 58). Developmental stage was scored according to the following classes: 1, morulae; 2, early blastocysts; 3, blastocysts; and 4, hatching or hatched blastocysts. **(B,C)** Representative images of morulae before vitrification and 24 h after warming, respectively.

There were no differences in embryo quality among the groups. The TCN (range: 50.5 ± 8.3–62.4 ± 10.3), TE (range: 37.7 ± 4.4–48.9 ± 12.0), and ICM (range: 12.7 ± 1.8–13.7 ± 1.8), as well as the ICM/TCN ratio (range: 20.6 ± 6.1–25.3 ± 5.0), were similar in the viable blastocysts, regardless of treatment (Figure 4). Apoptosis rates in morulae of the different experimental groups were also similar (range: 1.9 ± 2.1–3.4 ± 3.8%) (Figure 5). The cryotolerance of vitrified and warmed morulae in terms of postwarming survival (range: 81.5–87.7%) and embryonic developmental stage after 24 h of culture (range: 3.7 ± 0.4–3.9 ± 0.2) were also not affected by the treatments (Figure 6).

## Discussion

The combined short-term estrus synchronization and superovulation treatment used in this study had a negative effect on most of the reproductive parameters evaluated, particularly affecting the overall efficiency of pregnancy and embryo production.

In this experiment, we only used weaned sows of parity 3–7 because they are readily available on farms and because they are usually preferred in ET programs (3). In addition, unlike prepubertal and mature gilts (24–27), their response to superovulation treatment is very efficient in terms of embryo production and quality (11), which makes the use of these sows as embryo donors advisable. Moreover, sows of parity 1 and 2 not only have worse reproductive performance than sows of parity 3–7 (28–30) but also show remarkable differences in the interval between weaning and estrus, in the duration of estrus and the timing of ovulation (31–34), and in the response to Altrenogest treatment (35). We used an Altrenogest dose of 20 mg per sow per day, as lower doses (e.g., 16 mg) increased the incidence of ovarian cysts and polycystic ovaries (36), possibly due to inadequate treatment blockade of follicular growth (37, 38).

In the current experiment, as in other studies, treatment with Altrenogest successfully delayed estrus after weaning (14, 39, 40) and did not affect the percentage of sows that were in estrus within 10 days of treatment. However, the interval between Altrenogest withdrawal and estrus (SS groups) was longer than the interval between weaning and estrus (SC and C control groups), which is consistent with previous studies (14, 40). The reasons for the lengthening of this interval in sows treated with Altrenogest are unclear. At weaning, the resumption of ovarian activity (i.e., recruitment, development, and selection of ovarian follicles) is due to changes in the frequency and amplitude of GnRH/LH pulses in response to litter removal (41). In cycling sows, the drop in progesterone at the end of the luteal phase plays a key role in initial recruitment (42). Treatment with Altrenogest from the day of weaning blocks GnRH release and thus LH and FSH secretion (43), similar to the stimulus of sucking during lactation (41). The preceding inhibitory effects of lactation in combination with the administration of a progestogen at weaning may have exerted a higher inhibitory effect on the initiation of follicular recruitment and prolonged the interval to estrus, similar to the adverse effect of lactation catabolism or stress on the extension of weaning to the estrus interval (41, 44).

Our results clearly show that the combination of synchronization and superovulation treatments influences the occurrence of reproductive disorders. Overall pregnancy and embryo production efficiency in synchronized-superovulated sows were influenced by the presence of uterine infections and/or polycystic ovaries. In females treated with Altrenogest and eCG/hCG (SS groups), more than 13% of sows that had shown signs of estrus and more than 45% of sows that had not shown signs of estrus had uterine infections and/or polycystic ovaries. These results contrast with those observed in the control groups (SC and C groups), where no polycystic ovaries were observed and only 1.5 and 0.0% of sows with and without estrus, respectively, had uterine infections. In addition, the synchronization-superovulation treatment increased the percentage of sows with small ovarian cysts (~60%) and the mean number of these cysts per ovary (~3 cysts) twofold compared to the superovulated control and untreated control sows (~30% and ~1.5 cysts, respectively). These small cysts were present in one or in both ovaries, showed no signs of ovulation, and cohabited with normal corpora lutea. Since these data were similar between superovulated and untreated control sows, we confirmed our previous finding that single superovulation treatment at the dose used in weaned sows was not associated with the incidence of ovarian cysts (11). Furthermore, these small cysts, like the single cysts (45), had no effect on fertilization events or early embryo development, as they were unlikely to be functional, given the excellent fertilization and embryo production rates and embryo quality in sows from these control groups. In contrast, the combination of synchronization and superovulation treatments was involved in the development of small cysts and, more importantly, in the presence of polycystic ovaries, a pathology associated with infertility (46) that affected the overall pregnancy and embryo production efficiency.

These results were not unexpected. Polycystic ovaries have been associated with a low dosage of Altrenogest treatment in sows diagnosed as nonpregnant (36), and more recently, several studies indicated that treatment with Altrenogest in combination with exogenous gonadotropins causes the occurrence of a large number of ovarian follicular cysts in gilts (47, 48). Regardless of treatment, some sows (3.0–9.0%) that showed no signs of estrus within 10 days of treatment cessation or weaning were found to have normal ovaries and a good number of well-developed corpora lutea at laparotomy. Although this fact has been mainly attributed to inadequate management during estrus detection (49), it is more likely that, in our study, the main reason for the absence of typical estrus behavior was the presence of silent estrus (ovulation without visible signs of estrus) (50). In support of this speculation, our study was conducted in a production farm with more than 10,000 breeding sows, and the detection of estrus was performed in the presence of boars by the highly trained personnel of the farm.

Synchronization-superovulation treatment not only negatively affected pregnancy rates on day 6 of the cycle of sows inseminated during estrus but also decreased the overall pregnancy efficiency, fertilization rate and overall embryo production efficiency by more than 15 percentage points compared to superovulated control sows. Previous studies indicated that a short-term Altrenogest treatment (3–15 days), when administered independently, is effective for grouping the estrus of weaned primiparous and multiparous sows (14, 15, 40, 51–53). Moreover, Altrenogest treatment for 14–18 days improved the reproductive performance of gilts by increasing pregnancy and farrowing rates and litter size (35). On the other hand, superovulation with 1,000 IU eCG 24 h after weaning followed by a dose of 750 IU hCG at the beginning of estrus in sows not treated with Altrenogest markedly increased the number of corpora lutea, viable and transferable embryos and the *in vivo* developmental ability of the superovulated embryos without affecting the number of unfertilized oocytes (11, 12). The present study confirms these results and shows that superovulated control sows (SC group) had more corpora lutea and viable embryos and higher embryo production efficiency than the untreated control sows (C group). In addition, fertilization rates and the percentage of unfertilized oocytes were not affected by superovulation. Overall, these results suggest that the effects of combined synchronization and superovulation treatments on reproductive parameters are detrimental compared to those observed when these treatments are used independently. Although further study is needed to identify molecular changes at the ovary and genital tract levels after administration of this combined treatment, it could be speculated that its detrimental effects is related to a cumulative hormonal effect that affects ovarian and/or uterine functionality and influences oocyte maturation, sperm transport in the female reproductive tract, fertilization, and/or early embryonic development. In support of this speculation, our results confirm previous conjectures (54, 55) and show that synchronization-superovulation treatment increased the ovulatory response by releasing a large number of immature oocytes, which impaired fertilization. While nearly 70% of the structures classified as unfertilized oocytes or degenerated embryos were in fact immature oocytes in the synchronized-superovulated sows, no immature oocytes were observed in control sows. The presence of a high percentage of ovulated immature oocytes, which cannot be fertilized (56), in synchronized-superovulated sows is probably another cause of the reduced pregnancy and embryo production efficiency. Since the quality of the surviving day 6 embryos was similar among the groups, it could be speculated that events related to early embryo development were not involved in the negative effects of the synchronization-superovulation treatment.

## Conclusion

The results of this study show that the combination of Altrenogest synchronization treatment with superovulatory eCG treatment was effective in delaying post-weaning estrus and in inducing superovulation in weaned multiparous sows. However, this combined treatment negatively affected the interval to the onset of estrus, ovarian status, pregnancy rate, most of the reproductive parameters assessed, and the overall embryo production efficiency. In addition, our data confirm previous reports on the high efficiency of a single superovulation treatment with exogenous gonadotropins in multiparous sows after weaning.

## Data Availability Statement

The raw data supporting the conclusions of this article will be made available by the authors, without undue reservation.

## Ethics Statement

The animal study was reviewed and approved by the Ethics Committee for experiments with animals of the University of Murcia, Murcia, Spain (Code: 486/2018). Written informed consent was obtained from the owners for the participation of their animals in this study.

## Author Contributions

HR-M, IP, and EM contributed to conception and design of the study. CC, MG, IP, and EM directed the experiments. HG-R, CC, JC, AG-P, JMV, JLV, MG, AL-S, IP, and EM performed the experiments. CC, JMV, JLV, HR-M, IP, and EM performed the statistical analysis and analyzed and interpreted the data. HG-R, IP, and EM wrote the first draft of the manuscript. All authors contributed to manuscript revision, read, and approved the submitted version.

## Funding

This research was funded by the MCIN/AEI/ 10.13039/501100011033 and by ERDF a way of making Europe (RTI2018-093525-B-I00), Madrid, Spain; Fundacion Seneca (19892/GERM/15), Murcia, Spain; and the Swedish Research Council FORMAS (Projects 2017-00946 and 2019-00288), Stockholm, Sweden.

## Conflict of Interest

The authors declare that the research was conducted in the absence of any commercial or financial relationships that could be construed as a potential conflict of interest.

## Publisher's Note

All claims expressed in this article are solely those of the authors and do not necessarily represent those of their affiliated organizations, or those of the publisher, the editors and the reviewers. Any product that may be evaluated in this article, or claim that may be made by its manufacturer, is not guaranteed or endorsed by the publisher.

## References

[1] Martinez EA Martinez CA Nohalez A Sanchez-Osorio J Vazquez JM Roca J. Nonsurgical deep uterine transfer of vitrified, in vivo-derived, porcine embryos is as effective as the default surgical approach. Sci Rep. 2015;5(March):1–9. 10.1038/srep10587 26030839 PMC4450750

[2] Martinez EA Nohalez A Martinez CA Parrilla I Vila J Colina I. The recipients' parity does not influence their reproductive performance following non-surgical deep uterine porcine embryo transfer. Reprod Domest Anim. (2016) 51:123–9. 10.1111/rda.12654 26661993

[3] Nohalez A Martinez CA Reixach J Diaz M Vila J Colina I. Factors of importance when selecting sows as embryo donors. Animal. (2017) 11:1330–5. 10.1017/S1751731117000325 28219466

[4] Martinez CA Nohalez A Parrilla I Lucas X Sanchez-Osorio J Roca J. Simple storage (CO2-free) of porcine morulae for up to three days maintains the in vitro viability and developmental competence. Theriogenology. (2018) 108:229–38. 10.1016/j.theriogenology.2017.12.001 29253666

[5] Cuello C Martinez CA Nohalez A Parrilla I Roca J Gil MA. Effective vitrification and warming of porcine embryos using a pH-stable, chemically defined medium. Sci Rep. (2016) 6:33915. 10.1038/srep33915 27666294 PMC5036199

[6] Martinez CA Cambra JM Nohalez A Parrilla I Roca J Vazquez JL. Prevention of hatching of porcine morulae and blastocysts by liquid storage at 20 degrees C. Sci Rep. (2019) 9:6219. 10.1038/s41598-019-42712-x 30996298 PMC6470143

[7] Martinez EA Angel MA Cuello C Sanchez-Osorio J Gomis J Parrilla I. Successful non-surgical deep uterine transfer of porcine morulae after 24 hour culture in a chemically defined medium. PLoS ONE. (2014) 9. 10.1371/journal.pone.0104696 25118944 PMC4131926

[8] Dyck GW. Puberty, post-weaning estrus and estrous cycle length in yorkshire and lacombe swine. Can J Anim Sci. (1971) 51:135–40. 10.4141/cjas71-018

[9] Caárdenas H Pope WF. Control of ovulation rate in swine. J Anim Sci. (2002) 80:E36–46. 10.2527/animalsci2002.0021881200800ES10007x

[10] Martinez EA Cuello C Parrilla I Martinez CA Nohalez A Vazquez JL. Recent advances toward the practical application of embryo transfer in pigs. Theriogenology. (2016) 85:152–61. 10.1016/j.theriogenology.2015.06.002 26164803

[11] Angel MA Gil MA Cuello C Sanchez-Osorio J Gomis J Parrilla I. The effects of superovulation of donor sows on ovarian response and embryo development after nonsurgical deep-uterine embryo transfer. Theriogenology. (2014) 81:832–9. 10.1016/j.theriogenology.2013.12.017 24462299

[12] Hazeleger W Bouwman EG Noordhuizen JP Kemp B. Effect of superovulation induction on embryonic development on day 5 and subsequent development and survival after nonsurgical embryo transfer in pigs. Theriogenology. (2000) 53:1063–70. 10.1016/S0093-691X(00)00252-1 10798484

[13] Kraeling RR Webel SK. Current strategies for reproductive management of gilts and sows in North America. J Anim Sci Biotechnol. (2015) 6:3. 10.1186/2049-1891-6-3 25838898 PMC4382856

[14] Patterson J Wellen A Hahn M Pasternak A Lowe J DeHaas S. Responses to delayed estrus after weaning in sows using oral progestagen treatment. J Anim Sci. (2008) 86:1996–2004. 10.2527/jas.2007-0440 18407977

[15] van Leeuwen JJJ Williams SI Kemp B Soede NM. Post-weaning Altrenogest treatment in primiparous sows; the effect of duration and dosage on follicular development and consequences for early pregnancy. Anim Reprod Sci. (2010) 119:258–64. 10.1016/j.anireprosci.2010.02.008 20223607

[16] Pursel VG Johnson LA. Freezing of boar spermatozoa: fertilizing capacity with concentrated semen and a new thawing procedure. J Anim Sci. (1975) 40:99–102. 10.2527/jas1975.40199x 1110222

[17] Martinez CA Nohalez A Parrilla I Vazquez JL Roca J Cuello C. Surgical embryo collection but not nonsurgical embryo transfer compromises postintervention prolificacy in sows. Theriogenology. (2017) 87:316–20. 10.1016/j.theriogenology.2016.09.009 27707545

[18] Funahashi H Ekwall H Rodriguez-Martinez H. Zona reaction in porcine oocytes fertilized in vivo and in vitro as seen with scanning electron microscopy. Biol Reprod. (2000) 63:1437–42. 10.1095/biolreprod63.5.1437 11058549

[19] Martinez CA Nohalez A Ceron JJ Rubio CP Roca J Cuello C. Peroxidized mineral oil increases the oxidant status of culture media and inhibits in vitro porcine embryo development. Theriogenology. (2017) 103:17–23. 10.1016/j.theriogenology.2017.07.028 28763725

[20] Martinez CA Gil MA Parrilla I Martinez EA Cuello C. Protocol for porcine embryo transfer. part 1: embryos produced in vivo. In: Manual of the International Embryo Transfer Society. 5th Editio. Illinois USA: International Embryo Transfer Society 2441 Village Green Place Champaign. (2020) p. 3a1–9.

[21] Wydooghe E Vandaele L Beek J Favoreel H Heindryckx B De Sutter P. Differential apoptotic staining of mammalian blastocysts based on double immunofluorescent CDX2 and active caspase-3 staining. Anal Biochem. (2011) 416:228–30. 10.1016/j.ab.2011.05.033 21684250

[22] Brison DR Schultz RM. Increased incidence of apoptosis in transforming growth factor alpha-deficient mouse blastocysts. Biol Reprod. (1998) 59:136–44. 10.1095/biolreprod59.1.136 9675004

[23] Petters RM Wells KD. Culture of pig embryos. J Reprod Fertil Suppl. (1993) 48:61–73. 8145215

[24] Guthrie HD Henricks DM Handlin DL. Plasma hormone levels and fertility in pigs induced to superovulate with PMSG. J Reprod Fertil. (1974) 41:361–70. 10.1530/jrf.0.0410361 4452979

[25] Holtz W Schlieper B. Unsatisfactory results with the transfer of embryos from gilts superovulated with PMSG and hCG. Theriogenology. (1991) 35:1237–49. 10.1016/0093-691X(91)90369-O

[26] Wallenhorst S Holtz W. Embryo collection in prepubertal gilts and attempts to develop an improved embryo transfer technique. Vet Rec. (2002) 150:749–51. 10.1136/vr.150.24.749 12092622

[27] Ziecik A Biallowicz M Kaczmarek M Demianowicz W Riopérez J Wasielak M. Influence of estrus synchronization of prepubertal gilts on embryo quality. J Reprod Dev. (2005) 51:379–84. 10.1262/jrd.17008 15827382

[28] Koketsu Y Takahashi H Akachi K. Longevity, lifetime pig production and productivity, and age at first conception in a cohort of gilts observed over six years on commercial farms. J Vet Med Sci. (1999) 61:1001–5. 10.1292/jvms.61.1001 10535505

[29] Flowers WL Alhusen HD. Reproductive performance and estimates of labor requirements associated with combinations of artificial insemination and natural service in swine. J Anim Sci. (1992) 70:615–21. 10.2527/1992.703615x 1563988

[30] Hughes PE. Effects of parity, season and boar contact on the reproductive performance of weaned sows. Livest Prod Sci. (1998) 54:151–7. 10.1016/S0301-6226(97)00175-914643872

[31] Hurtgen JP Leman AD Crabo B. Seasonal influence on estrous activity in sows and gilts. J Am Vet Med Assoc. (1980) 176:119–23. 7353985

[32] Clark JR Komkov A Tribble LF. Effects of parity, season, gonadotropin releasing hormone and altered suckling intensity on the interval to rebreeding in sows. Theriogenology. (1986) 26:299–308. 10.1016/0093-691X(86)90149-4 16726195

[33] Koketsu Y Dial GD. Factors influencing the postweaning reproductive performance of sows on commercial farms. Theriogenology. (1997) 47:1445–61. 10.1016/S0093-691X(97)00135-0 16728090

[34] Soede NM Kemp B. Expression of oestrus and timing of ovulation in pigs. J Reprod Fertil Suppl. (1997) 52:91–103. 9602722

[35] Wang Z Liu BS Wang XY Wei QH Tian H Wang LQ. Effects of Altrenogest on reproductive performance of gilts and sows: A meta-analysis. Anim Reprod Sci. (2018) 197:10–21. 10.1016/j.anireprosci.2018.08.035 30197055

[36] Kauffold J Beckjunker J Kanora A Zaremba W. Synchronization of estrus and ovulation in sows not conceiving in a scheduled fixed-time insemination program. Anim Reprod Sci. (2007) 97:84–93. 10.1016/j.anireprosci.2006.01.004 16481132

[37] Martinat-Botté F Bariteau F Badouard B Terqui M. Control of pig reproduction in a breeding programme. J Reprod Fertil Suppl. (1985) 33:211–28. Available online at: http://europepmc.org/abstract/MED/3910826 3910826

[38] Soede NM Bouwman EG Langendijk P van der Laan I Kanora A Kemp B. Follicle development during luteal phase and Altrenogest treatment in pigs. Reprod Domest Anim. (2007) 42:329–32. 10.1111/j.1439-0531.2006.00779.x 17506814

[39] Wood CM Kornegay ET Shipley CF. Efficacy of Altrenogest in synchronizing estrus in two swine breeding programs and effects on subsequent reproductive performance of sows. J Anim Sci. (1992) 70:1357–64. 10.2527/1992.7051357x 1526904

[40] Santos JMG dos Wentz I Bortolozzo FP Barioni W. Early-weaned sows: Altrenogest therapy, estrus, ovulation, and reproductive performance. Anim Reprod Sci. (2004) 84:407–13. 10.1016/j.anireprosci.2004.02.011 15302382

[41] Shaw HJ Foxcroft GR. Relationships between LH, FSH and prolactin secretion and reproductive activity in the weaned sow. J Reprod Fertil. (1985) 75:17–28. 10.1530/jrf.0.0750017 3928882

[42] Soede NM Langendijk P Kemp B. Reproductive cycles in pigs. Anim Reprod Sci. (2011) 124:251–8. 10.1016/j.anireprosci.2011.02.025 21397415

[43] Stevenson JS Davis DL Pollmann DS. Altrenogest and fat for summer breeding of primiparous sows. J Anim Sci. (1985) 61:480–6. 10.2527/jas1985.612480x 4044446

[44] van den Brand H Dieleman SJ Soede NM Kemp B. Dietary energy source at two feeding levels during lactation of primiparous sows: I. Effects on glucose, insulin, and luteinizing hormone and on follicle development, weaning-to-estrus interval, and ovulation rate. J Anim Sci. (2000) 78:396–404. 10.2527/2000.782396x 10709931

[45] Tummaruk P Kesdangsakonwut S. Factors affecting the incidence of cystic ovaries in replacement gilts. Comp Clin Path. (2012) 21:1–7. 10.1007/s00580-010-1055-9

[46] Heinonen M Leppävuori A Pyörälä S. Evaluation of reproductive failure of female pigs based on slaughterhouse material and herd record survey. Anim Reprod Sci. (1998) 52:235–44. 10.1016/S0378-4320(98)00105-5 9783996

[47] Ziecik AJ Klos J Przygrodzka E Milewski R Jana B. Aberrant effects of Altrenogest and exposure to exogenous gonadotropins on follicular cysts appearance in gilts. Theriogenology. (2017) 89:250–4. 10.1016/j.theriogenology.2016.10.028 28043359

[48] Ziecik A Drzewiecka K Gromadzka-Hliwa K Klos J Witek P Knapczyk-Stwora K. Altrenogest affects the development and endocrine milieu of ovarian follicles in prepubertal and mature gilts. Biol Reprod. (2020) 103:1069–84. 10.1093/biolre/ioaa136 32744329

[49] Kauffold J Peltoniemi O Wehrend A Althouse GC. Principles and clinical uses of real-time ultrasonography in female swine reproduction. Anim an open access J from MDPI. (2019) 9. 10.3390/ani9110950 31717951 PMC6912286

[50] Althouse GC Kauffold J Rossow S. Diseases of the reproductive system. Diseases of Swine. (2019) 373–92. 10.1002/9781119350927.ch2025855820

[51] Kitkha S Boonsoongnern A Ratanavanichrojn N Jirawattanapong P Pinyopummin A. Effect of dosage and duration of Altrenogest treatmenton follicular development and ovulation in sows. Turkish J Vet Anim Sci. (2017) 41:733–40. 10.3906/vet-1703-54

[52] Lopes TP Bolarín A Martínez EA Roca J. Altrenogest treatment before weaning improves litter size in sows. Reprod Domest Anim. (2017) 52 Suppl 4:75–7. 10.1111/rda.13063 29052320

[53] Corezzolla J Ulguim R Gasperin B Rauber L Bianchi I. Altrenogest treatment effects on the reproductive performance of sows during transition to batch farrowing. Ciência Rural. (2020) 1:50. 10.1590/0103-8478cr20190806

[54] Estienne MJ Harper AF Horsley BR Estienne CE. Knight JW. Effects of PG 600 on the onset of estrus and ovulation rate in gilts treated with Regu-mate. J Anim Sci. (2001) 79:2757–61. 10.2527/2001.79112757x 11768102

[55] Horsley BR Estienne MJ Harper AF Purcell SH Baitis HK Beal WE.. Effect of PG 600 on the timing of ovulation in gilts treated with Altrenogest. J Anim Sci. (2005) 83:1690–5. 10.2527/2005.8371690x 15956478

[56] Hunter RHF. The effect of superovulation on fertilisation and embryonic survival in the pig. Anim Sci. (1966) 8:457–65. 10.1017/S000335610003813730886898

